# 3D-Bioprinted Gelatin Methacryloyl-Strontium-Doped Hydroxyapatite Composite Hydrogels Scaffolds for Bone Tissue Regeneration

**DOI:** 10.3390/polym16131932

**Published:** 2024-07-06

**Authors:** Cosmin Iulian Codrea, Dilruba Baykara, Raul-Augustin Mitran, Ayşe Ceren Çalıkoğlu Koyuncu, Oguzhan Gunduz, Anton Ficai

**Affiliations:** 1 Faculty of Chemical Engineering and Biotechnologies, National University of Science and Technology Politehnica of Bucharest, 060042 Bucharest, Romania; ccodrea@icf.ro; 2 Institute of Physical Chemistry “Ilie Murgulescu” of the Romanian Academy, 060021 Bucharest, Romania; raul.mitran@gmail.com; 3 Center for Nanotechnology & Biomaterials Application and Research (NBUAM), Marmara University, 34722 Istanbul, Turkey; baykaradilruba@gmail.com (D.B.); aceren@marmara.edu.tr (A.C.Ç.K.); 4 Department of Metallurgical and Materials Engineering, Faculty of Technology, Marmara University, 34722 Istanbul, Turkey; 5 National Research Center for Micro and Nanomaterials, Faculty of Chemical Engineering and Biotechnologies, National University of Science and Technology POLITEHNICA Bucharest, 060042 Bucharest, Romania; 6 National Centre for Food Safety, National University of Science and Technology POLITEHNICA Bucharest, 060042 Bucharest, Romania; 7 Academy of Romanian Scientists, Ilfov St. 3, 50044 Bucharest, Romania

**Keywords:** hydroxyapatite, precipitation, hydrothermal, strontium, gelatin methacryloyl, 3D-printing, digital light processing, bioactivity

## Abstract

New gelatin methacryloyl (GelMA)—strontium-doped nanosize hydroxyapatite (SrHA) composite hydrogel scaffolds were developed using UV photo-crosslinking and 3D printing for bone tissue regeneration, with the controlled delivery capacity of strontium (Sr). While Sr is an effective anti-osteoporotic agent with both anti-resorptive and anabolic properties, it has several important side effects when systemic administration is applied. Multi-layer composite scaffolds for bone tissue regeneration were developed based on the digital light processing (DLP) 3D printing technique through the photopolymerization of GelMA. The chemical, morphological, and biocompatibility properties of these scaffolds were investigated. The composite gels were shown to be suitable for 3D printing. In vitro cell culture showed that osteoblasts can adhere and proliferate on the surface of the hydrogel, indicating that the GelMA-SrHA hydrogel has good cell viability and biocompatibility. The GelMA-SrHA composites are promising 3D-printed scaffolds for bone repair.

## 1. Introduction

Treatment of bone diseases has an important clinical and economic impact, being known that ~50% of the total market share of the grafts is associated with the bone grafts. Bone is one of the most transplanted tissues, with an unmet ongoing demand that cannot be covered by naturally occurring allo-, auto-, and xenografts. There is, therefore, a strong focus on designing new materials with controllable properties capable of stimulating bone tissue regeneration. Research on osteogenic biomaterials is continuously looking for more efficient, cost-effective, and easy-to-produce materials. Successful bone repair materials need to be biocompatible, biodegradable, and similar in strength to bone tissue while allowing for good bone induction and having a 3D porous network structure for host tissue ingrowth [1]. Although the research literature is abundant in reports on 3D bioprinting of bone-like structures, the ideal solution for bone tissue regeneration has yet to be discovered. In fact, the material design is recognized worldwide to be of great potential, and this is especially true because of advances in both additive manufacturing and computer-tomography capabilities, which can also allow a very good mimicking of the natural bone structure. On the other hand, advanced 3D printing can tune the diffusion characteristics, the water uptake, and the release profile of the desired active agents.

Osteoporosis is a systemic bone mass-reducing illness with a risk of bone fragility, fractures, and long-lasting negative effects, such as reduced bone formation, delayed post-fracture healing, and impaired biomechanical properties [2]. Interventions such as bone transplantation, bone cement, or medication are necessary to increase bone mass, as the bone self-healing process is slow and inconsistent [3]. Anti-osteoporotic drugs, including estrogen, calcitonin, bisphosphonates, raloxifene, and RANK ligand inhibitors, already exist on the market, but their use is restricted due to side effects and high costs. Systemic high doses and frequent administration due to their low bone-targeting efficiency further complicate patients’ well-being with serious side effects [4].

Hydroxyapatite (HA) nanoparticles (Ca_10_(PO_4_)_6_(OH)_2_) play an important role in bone tissue regeneration due to their similarity to the inorganic component of the native bone [5]. HA possesses excellent biocompatibility [6], affinity to biopolymers, high osteogenic potential, and new bone in-growth-promoting properties through osteoconduction without toxicity or immune response [7]. Bone apatite is the crucial component of hard tissues, making up 60–70% of bone mass and 98% of the dental enamel mass [8]. HA can promote the adhesion, proliferation of osteoblasts, and extracellular matrix secretion while forming chemical bonds with the native bone tissue [3]. HA has the ability to adsorb and release therapeutic substances [9], making it an advantageous biomaterial for drug delivery systems [5]. Strontium-substituted HA (SrHA) is considered an established platform for the release of Sr ions, which are known to enhance bone formation and remodeling [8]. SrHA is also compatible with the polymers typically used for the design of bone-repair composite materials [10].

Hydrogels consist of a hydrophilic polymer scaffold with controllable mechanical properties that can simulate the natural extracellular matrix of bones and provide an adequate environment for endogenous cell growth [3]. Gelatin methacryloyl (GelMA) is a methacrylic anhydride-modified gelatin used as a base for 3D printing hydrogel bioinks. GelMA can be covalently cross-linked under UV light [1] and methacryloyl groups in the presence of a photoinitiator. GelMA forms 3D structures with customized geometry for implantation [3] and controllable mechanical properties capable of matching the requirements for scaffolds under diverse conditions [11]. These hydrogels exhibit many beneficial biological features, and they are being investigated for numerous applications ranging from drug delivery to tissue engineering [12]. GelMA has the advantage of its natural Arg-Gly-Asp (RGD) sequences, which can facilitate biological interaction between cells and scaffolds [11].

GelMA has limitations in bone defect regeneration due to low mechanical strength and lack of osteogenic factors and mineral ions. These drawbacks can be alleviated by the incorporation of inorganic fillers, which improve their mechanical properties and bone regenerative ability [13]. There are several reports on GelMA-gelatin bioink with the addition of HA for bone tissue engineering applications. Allen et al. demonstrated the suitability of a GelMA-gelatin-HA bioink for 3D bioprinting [1]. The addition of HA to GelMA-gelatin hydrogels significantly decreased hydrogel swelling, improved ability of the hydrogel to resist enzymatic degradation, increased osteoblastic differentiation and mineralization, and increased osteogenic gene expression while maintaining equal cell viability and proliferation to non-HA hydrogels [1]. Wang et al. proposed a biomimetic HA nanofibers/GelMA hydrogel for bone tissue engineering, showing that the incorporation of biomimetic SrHA improved the biocompatibility and the mechanical, swelling, degradation, and bone regenerative performances of GelMA [13]. GelMA bioink, containing HA nanoparticles and cells, was successfully prepared for direct 3D bioprinting of cell-laden bone matrices by Zhou et al. [14]. Wang et al. studied GelMA-PEGDA (poly (ethylene glycol) diacrylate)-HA nanoparticles ternary composite hydrogel materials with stronger mechanical properties, longer degradation time, lower swelling ratio, and better biocompatibility than pristine GelMA. These effects were explained by the incorporation of HA nanoparticles into the prepared material and the stabilizing effects of the calcium ions, which form Ca^2+^ hydroxyl bridges between HA and GelMA [3].

Liu et al. have designed and successfully constructed the GelMA/HA-based tri-layered scaffolds for repairing the more complex defects involving cartilage and subchondral bone. The scaffolds contained a GelMA-based top cartilage layer, an interfacial layer, and a bottom subchondral bone layer, the latter two containing 3% (*w*/*v*) nano-HA for improved osteoconductivity [15]. Leu Alexa et al. constructed 3D composite hydrogels based on GelMA reinforced with HA doped with Zn^2+^ and Mg^2+^ as potential scaffolds for bone tissue engineering [16]. Also, Chen et al. employed stereolithography to print GelMA-HA scaffolds [17]. However, previous studies have not addressed the urgent problem of addressing fractures associated with specific diseases, such as osteoporosis, and to try to develop materials with local Sr^2+^ release capacity.

3D bioprinting of GelMA-SrHA composite hydrogel scaffolds for bone tissue regeneration was proposed as a promising possibility in the treatment of critical-sized bone defects. It is suggested that Sr-containing biomaterials with osteoimmunomodulatory functions will constitute the base for designing the next generation of orthopedic implants [18]. The emergent qualities of the proposed composites qualify it as a suitable candidate for future bone implants because of the individual properties of its components, such as bone regeneration in the case of SrHA and drug delivery in the case of GelMA. Even though we found one previous attempt of using K^+^/Sr^2+^/Na^+^ triple-doped hydroxyapatite/GelMA composite hydrogel scaffold for the repair of bone defects [19], to the best of our knowledge, the use of Sr as an active anti-osteoporotic agent coupled with GelMA hydrogel scaffolds has never been studied for 3D printing of bone repair composites. Furthermore, a supplementary comparison was proposed on the effects that different SrHA synthesis methods have on the composite scaffold properties. The digital light processing (DLP) 3D printing technique was used in this study to produce GelMA—strontium-doped hydroxyapatite composites. The DLP technique uses a programmable light source whose emission, in terms of intensity, field distribution, and wavelength, are modulated. This modulation leads to fast printing speed, great scalability, and mild working conditions [20].

## 2. Materials and Methods

### 2.1. Materials

Gelatin (Type A, gel strength ~300 g Bloom, molecular weight 50–100 kDa), methacrylic anhydride (MAA), lithium phenyl-2,4,6-trimethyl-benzoyl phosphinate (LAP), and dialysis membrane (cut-off value 14 kDa and average flat width 43 mm) were purchased from Sigma–Aldrich (Darmstadt, Germany). Of sodium carbonate, sodium hydroxide, and hydrochloric acid, 37% were purchased from Merck KGaA (Darmstadt, Germany). Sodium hydrogen carbonate (>99.7%) was bought from ISOLAB (Eschau, Germany). Phosphate-buffered saline (PBS, pH 7.4) and Tris buffer (pH 7.6) were obtained from ChemBio (Bağcılar, Turkey).

### 2.2. Synthesis of Gelatin Methacryloyl (GelMA)

The synthesis was performed according to our previous literature report [21]. GelMA was prepared using 10% (*w*/*v*) solution of type A gelatin (Gelatin from porcine skin, Sigma, G1890, SLCB3384) in CB buffer (0.1 M CB buffer containing 3.18 g sodium carbonate and 5.86 g sodium bicarbonate in 1 L of distilled water, pH 9), at constant conditions (pH 9 and temperature of 60 °C). Methacrylic anhydride (MAA) was added to the gelatin solution in a proportion of 0.1 mL per gram of gelatin and allowed to react for 3 h at 50 °C under constant stirring. The reaction was then terminated by adjusting the pH to 7.4. The obtained solution was dialyzed through a 14 kDa molecular-weight-cutoff (MWCO) membrane in distilled water for 3 days at a constant temperature of 37 °C. Dialysis of GelMA solution helped to remove unreacted MAA and methacrylic acid byproducts. After the dialysis, the prepared solution was lyophilized for 3 days and stored at +4 °C until use.

### 2.3. Design and Manufacturing of 3D Composite Scaffolds

Computer-aided design (CAD) files containing the instructions for the scaffolds were designed using SolidWorks 2020 (Dassault Systèmes SE, Vélizy-Villacoublay, France), converted to the .stl file format and sliced using Chitubox (Shenzhen Chuangbide Technology Co., Ltd., Shenzhen, China), the software of the DLP-based 3D printer (Photon D2 DLP Printer, Anycubic, Shenzhen, China). Scaffolds were designed to be 18 × 18 × 1.3 mm in size and containing 3 × 3 macropores for the purpose of showing the printability capacities of the material.

HA and SrHA powders were synthesized using the precipitation and hydrothermal methods and exhaustively characterized, as presented in our previous literature report [22]. Molar ratios of Sr/(Ca + Sr) between 0 and 10% were used, and samples were denoted as HAPR and HAPR-Sr10% and HAHT and HAHT-Sr10% for the precipitation and hydrothermal method, respectively. The presence of Sr in the powders was highlighted using Electron energy loss spectroscopy (EELS) and quantified using Energy Dispersive X-ray (EDX), while the particle size of the powders was determined to be in the nano range using Scanning Electron Microscopy (SEM) and Transmission Electron Microscopy (TEM), as presented in our previous literature report [22]. Details about the particle size distribution can be found in Figures S1, S2 and Table S1 (Supplementary Materials). The photocurable printing material was prepared using a 10% (*w*/*v*) GelMA in PBS solution under constant stirring at 40 °C for 30 min. The photoinitiator (LAP) was added to the mixture at a 0.5% concentration and stirred for 10 min. HA and SrHA were added also in a 5% (*w*/*v*) concentration to the mix and stirred for another 10 min.

**Figure 1:**
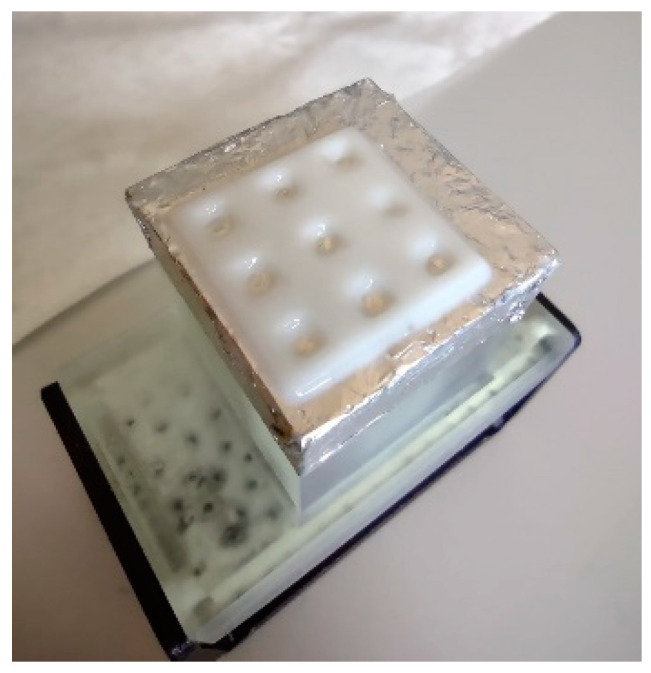
DLP printer component with a printed scaffold.

Subsequently, the mixture was brought to room temperature (~25 °C) and transferred to the tank of the DLP printer. DLP 3D printers use a facile photopolymerization process to cross-link GelMA and obtain the desired scaffold. Printing parameters were set to 12 mW/cm^2^ light intensity, 405 nm light wavelength, and an exposure time of 10 s, similar to the procedure used in another previous literature report [21]. The obtained 3D-printed scaffolds were dried in the dark at room temperature for 24 h and kept in a dehumidified container prior to characterization (Figure 1).

**Figure 2:**
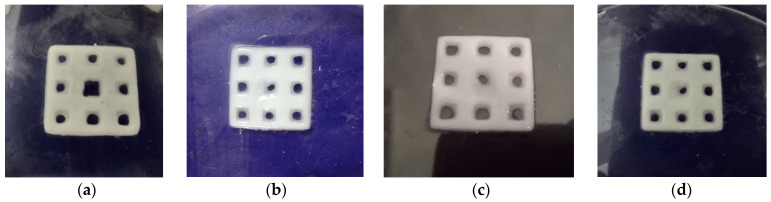
DLP obtained scaffolds: (**a**) GelMA-HAPR, (**b**) GelMA-HAHT, (**c**) GelMA-HAPR-Sr10%, (**d**) GelMA HAHT-Sr10%.

Four types of composite scaffolds were obtained: GelMA-HAPR, GelMA-HAHT, GelMA-HAPR-Sr10%, and GelMA-HAHT-Sr10% (Figure 2). Scaffolds containing only GelMA were also printed to be used as control samples. Samples were lyophilized before further analyses and use.

### 2.4. Characterization Methods for GelMA and Composite Scaffolds

The chemical structure of gelatin, GelMA, HAPR, HAPR-Sr10% HAHT, and HAHT-Sr10% was investigated using FTIR (FT/IR-ATR 4700 spectrometer, Jasco, Easton, MD, USA) at room temperature. Spectra were obtained in the 450 and 4000 cm^−1^ range at a resolution of 4 cm^−1^.

Thermogravimetric analyses (TGA) were carried out using a Mettler Toledo TGA/SDTA851e thermogravimeter at a heating rate of 10 °C min^−1^, under 80 mL min^−1^ synthetic air flow. All mass loss weight fractions were computed with respect to the dry sample mass, taken at 150 °C.

Hydrogel scaffolds were investigated using a scanning electron microscope (EVA MA 10, Zeiss, Jena, Germany) to evaluate the surface morphology of the 3D-printed square samples. Prior to analysis, the samples were lyophilized for one day and stored at +4 °C until use. Subsequently, the surfaces of the scaffolds were coated with gold using a spray coating machine (SC7620, Quorum, Laughton, East Sussex, UK) for 120 s. Average pore diameters (Feret’s diameter) were determined from SEM images using ImageJ software (National Institutes of Health, Bethesda, MD, USA).

The samples were put in 1 mL of PBS (pH 7.4) (Sigma–Aldrich) and incubated at 37 °C for different times (0.5, 1, 1.5, 2, 3, 4, 5, 6, and 24 h) after freeze-drying. The samples were then removed from PBS (pH 7.4), blotted with filter paper, and weighed. The swelling degree (*SD*) was calculated with the following formula (1):

(1) $$SD\;\left(\%\right)=\left(\frac{W_{w}-W_{d}\;}{W_{d}}\right)\times 100$$

where *W_w_* is the wet weight after swelling and *W_d_* the dry weight. The swelling capability was calculated to understand the absorption rate, especially the time needed for the sample to reach the maximum water uptake. Water uptake is important in all medical applications because it mediates the exchanges with the surrounding tissues and fluids.

The GelMA and the composite samples were freeze-dried and weighed. The dry composite was added to 1.5 mL of PBS (pH 7.4) and placed into a shaker. The temperature was set to 37 °C, and the rotation speed was 120 rotations per minute. The solution in the centrifuge tube was removed, and the sample was washed twice, then freeze-dried and weighed again at predetermined time intervals. The degradation degree (*DD*) was calculated with the following formula (2):

(2) $$DD\;\left(\%\right)=\left(\frac{W_{i}-W_{f}}{W_{f}}\right)\times 100$$

where *W_i_* is the initial dry weight of the samples and *W_f_* is the final dry weight.

Scaffolds were evaluated regarding their compression stiffness and mechanical strength using a compression testing machine (EZ-LX, Shimadzu, Kyoto, Japan). Cylindrical specimens of GelMA (8 mm in diameter and 6 mm in height) were used in the compression stiffness analysis. A rate of 1 mm/min and a maximum strain of 60% were used for this evaluation. Compressive modulus values were calculated from the initial linear region (0–20% of strain) of the obtained stress–strain curves. Scaffolds were placed on a stainless-steel plate, and an axial force was applied at a constant rate of 0.1 mm/min perpendicular to the axis of the samples for the mechanical strength analysis.

Cell viability assay was performed on cell-laden scaffolds. Scaffolds were first sterilized in a class II A2 biosafety cabinet (Demair, Turkey) under UV (at 254 nm wavelength) for 30 min, and then they were seeded with 5000 human fetal osteoblasts (HOB) cells/scaffold. On the first, 4th, and 7th days of culture, cell proliferation was analyzed by using MTS assay (Elabscience, Houston, TX, USA) according to the standard protocol. Briefly, 100 µL of culture media was mixed with 50 µL of MTS for each sample. After 2.5 h of incubation, the supernatants were discarded, and the formazan crystals on each well were dissolved with 150 µL of DMSO. Optical density values were measured at 570 nm with a microplate spectrophotometer (BioTek Epoch2, Agilent, Santa Clara, CA, USA). OD 570 values were converted to cell numbers by using a calibration curve for HOB.

The experiments were carried out in duplicate or triplicate, and data are expressed as mean ± standard deviation (SD). Post-hoc one-way ANOVA with a Tukey HSD Test was employed for statistical analysis. Statistical analysis was performed using MS Excel software (Microsoft, Redmond, WA, USA). Values of * *p* < 0.05 and ** *p* < 0.01 are considered statistically significant.

## 3. Results

A DLP 3D printer was employed to produce a series of bone matrices consisting of GelMA hydrogel with nanocrystalline HA/SrHA. 3D composite hydrogels based on GelMA were reinforced with HA and Sr-doped HA as potential scaffolds for bone tissue engineering. The 3D structure of composite scaffolds with four different types of ceramic HA and SrHA was achieved through 3D printing. The mechanical stability of scaffolds was improved through GelMA UV-chemical crosslinking. The physicochemical properties of GelMA-HA and GelMA-SrHA materials were determined as well as biocompatibility in vitro.

### 3.1. Fourier Transform Infrared Spectroscopy (FTIR)

**Figure 3:**
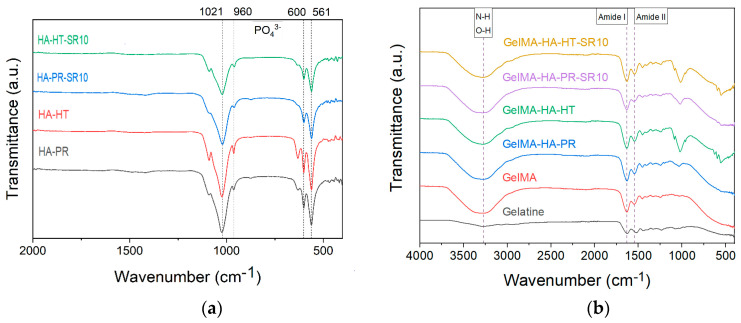
FTIR spectra of (**a**) HA-PR, HA-HT, HA-PR-Sr10, HA-HT-Sr10 and (**b**) gelatin, methacrylate gelatin, composite materials GelMA-HA-PR, GelMA-HA-HT, GelMA-HA-PR-Sr10, and GelMA-HA-HT-Sr10.

FTIR spectra of HA-PR and HA-HT (Figure 3a) show the characteristic functional groups of HA (PO_4_^3−^, HPO_4_^2−^) (the bending vibrations of v4 degenerate state at 561 and 600 cm^−1^, the symmetric v1 stretching vibration at 960 cm^−1^, and the asymmetric v3 stretching vibration at 1021 cm^−1^) [22,23]. SrHA has relatively broader peaks than HA due to the partial replacement of Ca^2+^ by Sr^2+^, which causes a crystal lattice expansion and less crystalline apatite phases [24].

The FTIR spectra also exhibit the specific absorption bands for the functional groups in the gelatin structure, between 1000 and 1700 cm^−1^. No changes in this range were noticed between gelatin and GelMA, indicating that in the methacrylation step, the bonds between the amino acids in the structure of the primary proteins were not affected [16]. The characteristic N-H and O-H stretching vibrations between 3200–3400 cm^−1^ can also be noticed as a broad signal (Figure 3b) [25,26]. Bands representative for amide I and II [17] at 1631 and 1529 cm^−1^ (owing to C=O stretching and N–H bending, respectively [25,27]) are also present in all samples.

### 3.2. Thermogravimetric Analyses (TGA)

**Figure 4:**
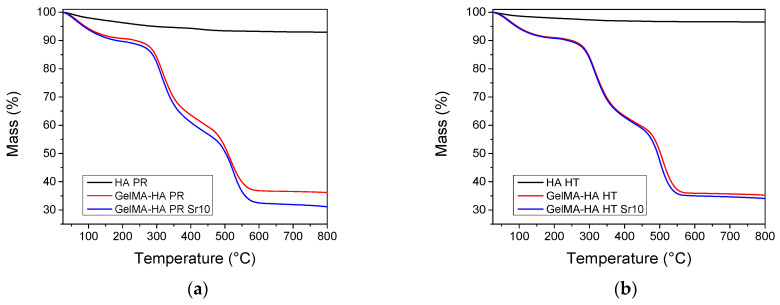
Thermogravimetric analyses of the samples containing (**a**) precipitation obtained HA/SrHA and (**b**) hydrothermal obtained HA/SrHA.

TGA was performed on HA samples and lyophilized GelMA-HA/SrHA composites. Both HA materials show a gradual mass loss when heating up to 800 °C, which could be ascribed to the loss of physisorbed water and hydroxyl groups (Figure 4). The composites containing GelMA exhibit three independent mass loss events [28]. Water desorption can be noticed on heating between 30 and 150 °C, followed by the combustion of the polymer in two distinct steps, between 200–400 °C and 400–600 °C. The composition of the samples was estimated from the mass loss at 650 °C. All weight fractions were computed with respect to the dry sample mass, experimentally determined at 150 °C. All samples contain 32.6–38.0% HA by weight (Table 1). The thermogravimetric analyses confirm the successful incorporation of HA inside the composite matrices.

### 3.3. Morphological Characterization

**Figure 5:**
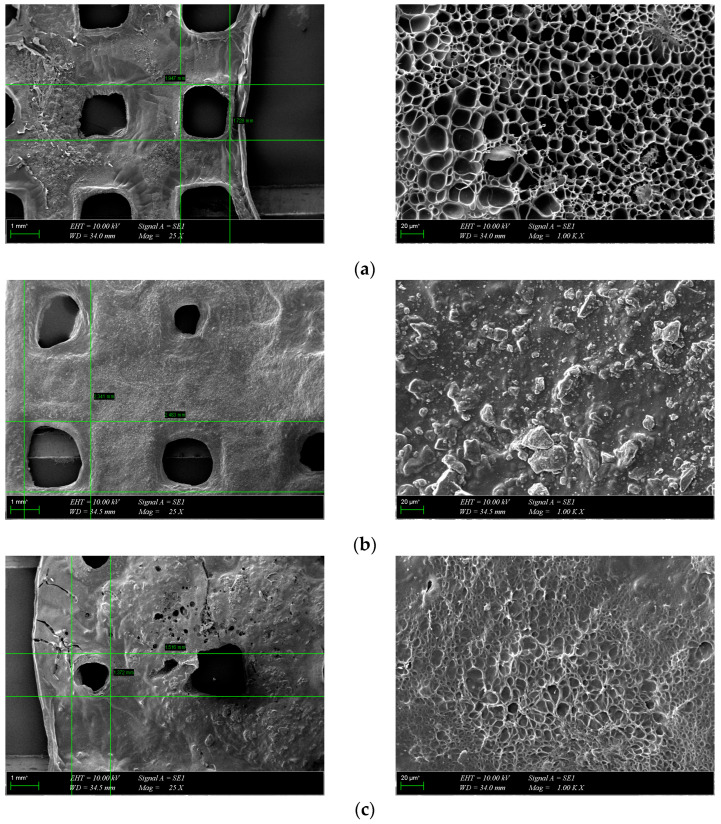

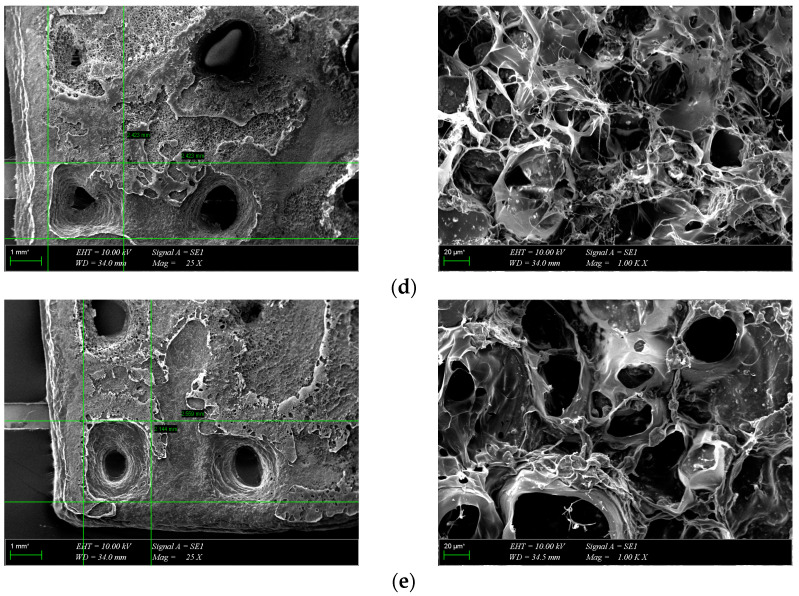
SEM images from the surface of GelMA and composite hydrogels: (**a**) GelMA, (**b**) GelMA-HA-PR, (**c**) GelMA-HA-HT, (**d**) GelMA-HA-PR-Sr10%, and (**e**) GelMA-HA-HT-Sr10%.

The morphology of the scaffolds is an important characteristic that plays a role in controlling their interaction with native tissue. Obtained hydrogel composites have an interconnected porous structure. This structure contributes to the retention of large amounts of water and promotes the diffusion of nutrients and macromolecules, an aspect beneficial to tissue regeneration [13]. The interconnected pores are beneficial for the migration and adhesion of a high cell density [16]. Figure 5 shows the 3D porous network structure useful for host tissue ingrowth. There is a significant difference between GelMA-HA-PR scaffolds (Figure 5b), which show no porous structure on the surface, and the other scaffolds. SEM images show the presence of HA/SrHA nanoparticles on the surface of the scaffolds. The HA particles present a uniform distribution inside the GelMA matrix. There is a significant difference between the GelMA-HA-PR scaffold and the other samples. For this sample, the particles of HA are clearly visible on the surface of the scaffold, and the surface looks more compact and rugged. The resemblance between the non-Sr-containing samples can be seen (Figure 5b,c), as well as between the Sr-containing samples (Figure 5d,e).

**Figure 6:**
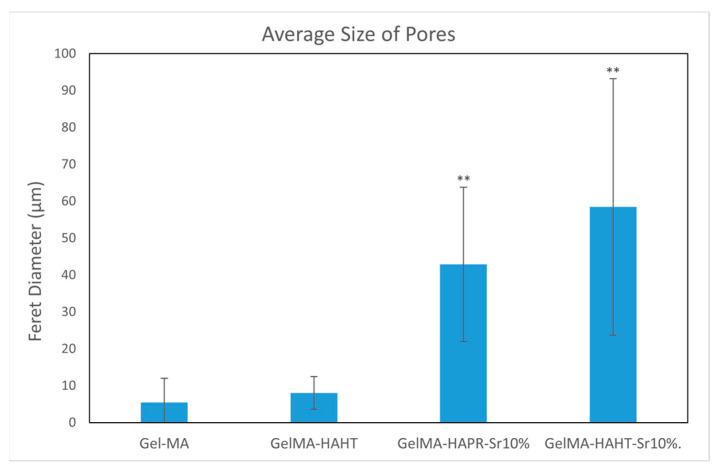
The average size of pores using Feret diameter measurements on GelMA and composite hydrogel samples. Results are represented as mean ± SD and relative to GelMA control samples (** *p* < 0.01 compared to control).

Pores were analyzed quantitatively using ImageJ. Average Feret diameters are presented in Figure 6, except for GelMA-HA-PR, which has no distinct pores and could not be analyzed through this method. Samples presented pore-shaped polydispersity. The average diameter is significantly higher in the case of Sr-containing samples. These measurements indicate that most of the pores observed on GelMA and GelMA-HA-HT are suited for cell adhesion, the average pore size being 55 μm in diameter, which is well fitting to allow the osteoblast to get inside these grafts and to assist the bone ingrowth [29]. GelMA-HA-PR-Sr10% and GelMA-HA-HT-Sr10% exhibit a large range of pore sizes, suggesting good cell adhesion, viability, and proliferation potential and corroborated with the SEM imaging; the rough surface will further enhance the adhesion of the cells.

### 3.4. Swelling Behavior

**Figure 7:**
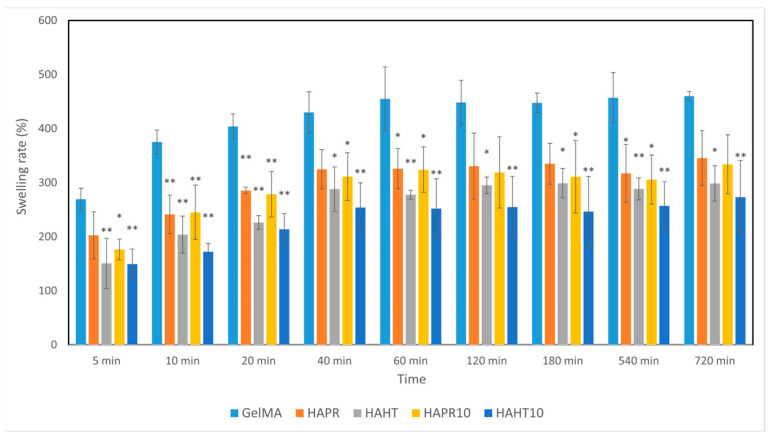
The swelling ability of GelMA and composite hydrogel scaffolds was incubated for different time intervals. Data are expressed as mean ± standard deviation (SD, n = 3) and relative to GelMA control samples (* *p* < 0.05 and ** *p* < 0.01 compared to control).

Hydrogels, which have a hydrophilic nature, increase in weight when they absorb water due to the presence of various functional groups (-NH_2_, -COOH, -OH, -CONH_2_, -CONH, and -SO_3_H) [30]. The swelling of hydrogels plays an important role in determining the hardness and porosity of the material [31]. Scaffolds were placed in PBS and then incubated to mimic the natural physiological conditions of living organisms. All composite scaffolds rapidly absorbed the PBS solution within 40 min and reached an equilibrium state. The swelling rates of GelMA (430%), GelMA-HAPR (324%), GelMA-HAPR-Sr10% (282%), GelMA-HAHT (295%), GelMA HAHT-Sr10 (254%) groups are shown in Figure 7. It was found that the swelling rate of the pure GelMA scaffolds was higher than that of the others. The results of the swelling behavior of composite scaffolds show that the addition of HA/SrHA to the scaffolds significantly reduces the swelling behavior. This is in accordance with Allen et al., who confirmed that the addition of HA to GelMA scaffolds decreased the swelling rate. This was hypothesized to be due to a mechanism by which HA limits the area that water can occupy in the hydrogel [1].

### 3.5. Degradation Performance

**Figure 8:**
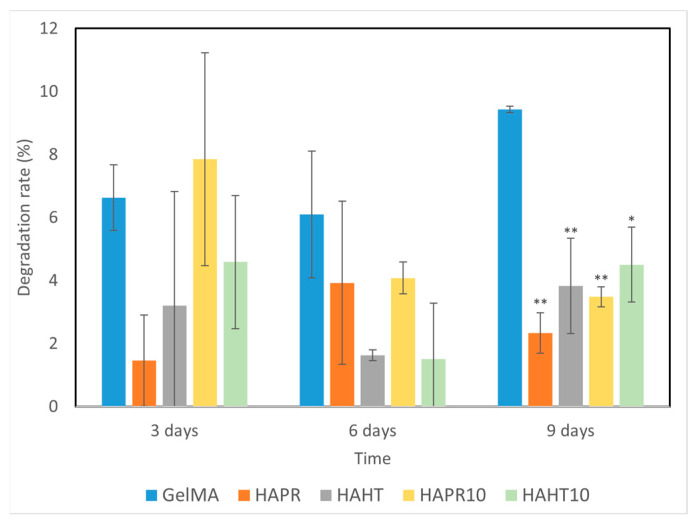
The degradation ability of GelMA and composite hydrogel scaffolds incubated for different time intervals. Data are expressed as mean ± standard deviation (SD, n = 2) and relative to GelMA control samples (* *p* < 0.05 and ** *p* < 0.01 compared to control).

Scaffolds, which are temporary substrates for the survival and growth of cells during the formation of new tissue, must have a controlled rate of degradation [32]. The degradation behavior of GelMA and composite scaffolds was evaluated by placing them in PBS solution, and the results are shown in Figure 8. The scaffold undergoes an ionic exchange with its surrounding medium, which probably implies the formation of an apatitic layer upon immersion of scaffolds into PBS. This possible apatite deposition may be the cause of the variations observed in the degradation rate of the composite scaffolds.

Overall, composite scaffolds had significantly lower degradation rates (1–8%) compared to the pristine GelMA (6–10%). Similarities occurred between the degradation patterns of GelMA-HAHT and GelMA-HAHT-Sr10%. A remarkably lower degradation rate was observed in the case of GelMA-HAPR, which may be due to the lower surface area of the scaffold, as noticed from the SEM micrographs (Figure 5b). The amide bond in the hydrogel can be coordinated by the Ca^2+^ in the HA/SrHA structure, thereby increasing the hydrogel’s stability. This characteristic, together with the natural stability of HA/SrHA in water solutions, allows the degradation rate of scaffolds to be adapted to particular requirements by increasing the HA/SrHA content in the structure [31]. In general, it can be affirmed that the degradation over 9 days is suitable for medical applications and bone fracture healing, considering that primary bone mass is obtained in several weeks.

### 3.6. Mechanical Analysis

It is important that the designed scaffold is biologically, chemically, and mechanically similar to the extracellular matrix to support tissue regeneration. The samples should have appropriate mechanical stability to support cell attachment, migration, and differentiation, but these properties can be easily transferred to temporary metallic implants, and, thus, the most important properties required from these materials are the biological and chemical ones [33]. In a study by Wang et al., nano-HA particles were added to GelMA hydrogel in different ratios. They concluded that the addition of nanoparticles increased the compressive strength [34]. Sr, which is added to stimulate osteogenesis, plays an important role in improving the biological properties of HA and facilitating the healing of bone defects [35].

**Figure 9:**
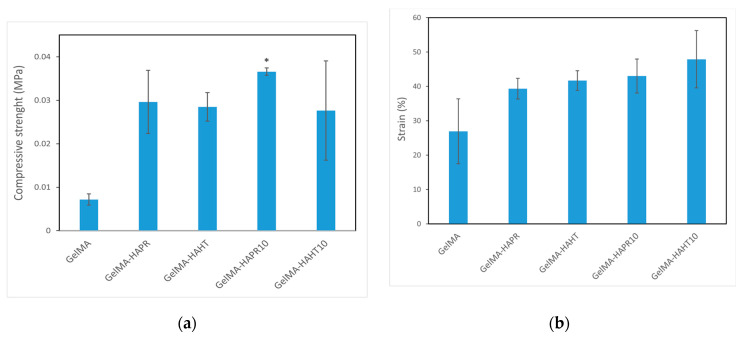
Mechanical properties of GelMA and composite hydrogels scaffolds (**a**) compressive strength, (**b**) strain (%). Data are expressed as mean ± standard deviation (SD, n = 2) and relative to GelMA control samples (* *p* < 0.05 compared to control).

The compressive strength of values of the GelMA-HA-PR (0.02962 ± 0.00727 MPa), GelMA-HA-HT (0.02849 ± 0.00329 MPa), GelMA-HA-PR-Sr10% (0.03659 ± 0.00085 MPa), and GelMA-HA-HT-Sr10% (0.02763 ± 0.01142 MPa) scaffolds were higher than that of GelMA (0.00718 ± 0.000131 MPa) (Figure 9a). The strain values (%) of the GelMA-SrHA composite scaffolds (Figure 9b) exhibit a similar increase in comparison to GelMA. The addition of HA/SrHA increased the mechanical stability and reduced the degradation rate of the hydrogel.

### 3.7. In Vitro Biological Evaluation of GelMA Scaffolds

**Figure 10:**
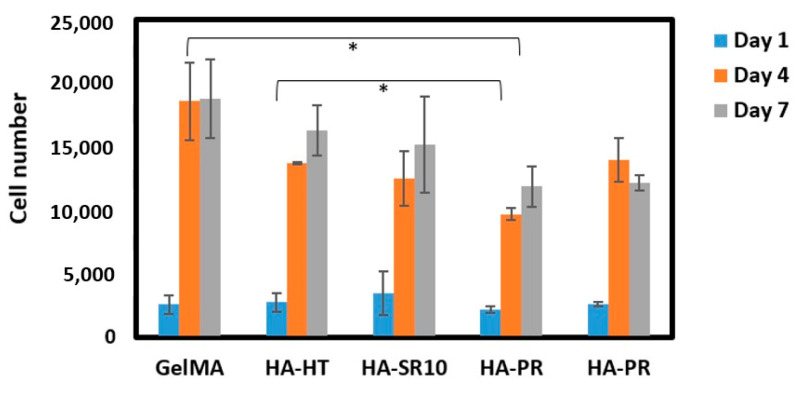
Cell viability on scaffolds at 1, 4, and 7 days of incubation (n = 3). * *p* values lower than 0.05 were shown on the graph.

The biocompatibility of the scaffolds was analyzed by performing MTS cell viability assay. Five thousand osteoblasts were seeded on the 3D-printed scaffolds, and then cell viability was measured after 1, 4, and 7 days of incubation. The cell proliferation on GelMA-HA-HT, GelMA-HA-HT-Sr10%, and GelMA-HA-PR scaffolds continued to increase on day 4 and day 7 (Figure 10). The cell number in GelMA-HA-HT scaffolds was significantly higher than the cell number in HA-PR samples (*p* < 0.05), both on day 4 and day 7. Cell proliferation in GelMA-HAHT-Sr10% scaffolds increased by more than fourfold on day 4 and continued to increase on day 7, while cell viability in GelMA-HAPR-Sr10% scaffolds increased by fivefold on day 4, but a small decrease was noticed on day 7, which was not statistically significant. Overall, there are significant differences between the results of cell proliferation testing when hydrothermal and precipitation samples were compared. Samples containing hydroxyapatite prepared by the hydrothermal route have higher cell proliferation rates than the samples containing HAPR. The synthesized HA-PR powder has a smaller particle size and a spherical shape, as described in our previous report [22]. It is, therefore, more prone to aggregation, behaving like larger particles. Li et al. mention the closeness of nanorod particles obtained through the hydrothermal method to those of the biological HA in natural bone [36]. The rod-shaped particles obtained in their study exhibited excellent biocompatibility. Our findings match those previously reported by Li et al. through the better results obtained for the scaffolds containing rod-shaped hydrothermally obtained HA/SrHA. This explains why the viability of the HA-PR and HA-PR-Sr10% containing scaffolds was lower than in the HA-HT and HA-HT-Sr10% ones. It is considered that Sr influences the differentiation and proliferation of osteoblasts, stimulating the secretion of bone matrix, but no significant difference was observed when comparing the Sr-containing scaffolds with the other composite scaffolds, probably due to the small quantity of Sr used in this study. To achieve more definite results, Sr could be added to the scaffolds in larger amounts. Overall, MTS assay results show relatively low cytotoxicity values in all samples, indicating high biocompatibility.

**Figure 11:**
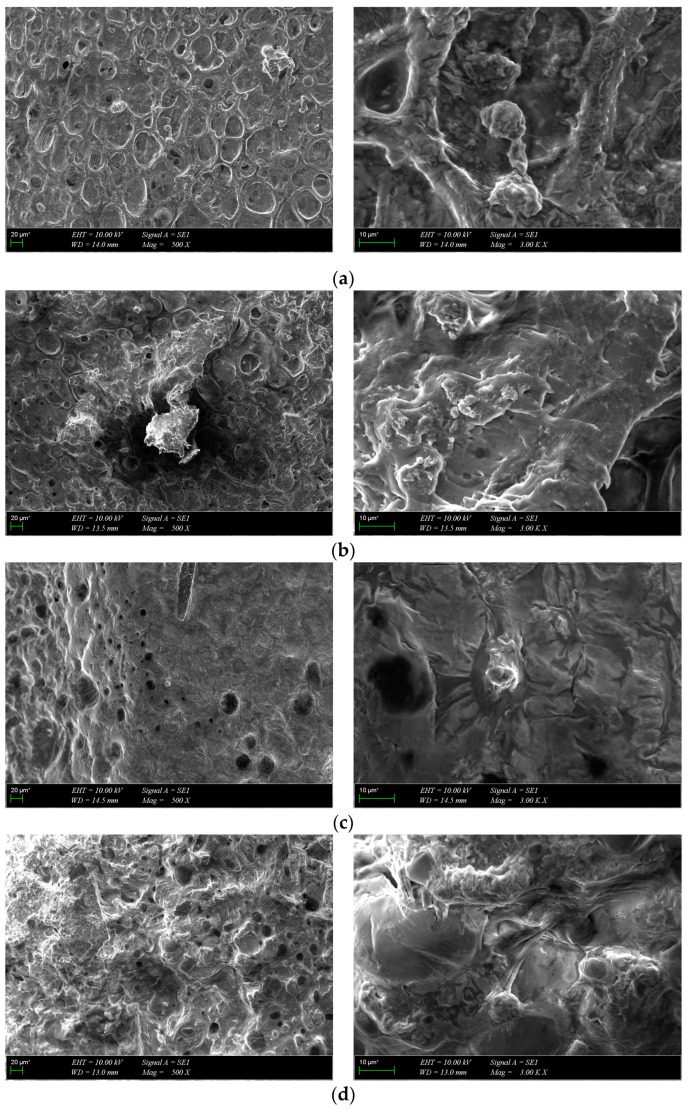
SEM images of cultured composite scaffolds on the 7th day of cell growth: (**a**) GelMA-HA-PR, (**b**) GelMA-HA-HT, (**c**) GelMA-HA-PR-Sr10%, (**d**) GelMA-HA-HT-Sr10%.

SEM images of osteoblast cells on scaffolds at different magnifications were acquired after 7 days of incubation (Figure 11). The cells showed strong adhesion to the surface of the scaffolds during the one-week culture period and spread out and covered the surface in the form of a layer. The images show that all scaffolds provided a favorable environment for cell growth, proliferation and migration. Collectively, MTS and SEM results suggest that the scaffolds were biocompatible with osteoblasts and promoted cell attachment and proliferation during the 7-day incubation period.

## 4. Conclusions

GelMA bioink, containing HA/SrHA, was successfully prepared for 3D bioprinting of bone regeneration scaffolds. The results indicated that the incorporation of nanosized HA/SrHA improved the mechanical, swelling, and degradation performances of GelMA. However, the improvement in the performance of the composite hydrogel did not increase in the same manner for every type of hydroxyapatite. Sr-containing scaffolds exhibited better swelling and biocompatibility performances compared with the non-Sr-containing group of scaffolds, while scaffolds containing hydrothermally obtained HA/SrHA had overall better results than scaffolds containing precipitation obtained HA/SrHA. GelMA-HA-HT-Sr10% gave the best overall results in terms of mechanical, swelling, and degradation performance.

Although good results were obtained, our study also had important limitations. Achieving high resolution and precision was limited by the in-built performances of the 3D printer. As we demonstrated, the fidelity with the CAD model can be obtained if requirements are not high even with a low-cost consumer-grade 3D printer, but the necessity of having a multi-scale architecture capable of stimulating both short-term cell adhesion and proliferation and long-term angiogenesis is unquestionable. Further studies are needed to investigate the capacity of the used composite to be printed in a scaffold with a more practical shape and a more complex architecture, necessary in the angiogenesis process, with the help of more powerful 3D printing equipment. Also, a better understanding of the long-term stability of the scaffolds, both in vitro and in vivo, is essential before any scaling up or regulatory approval perspectives can be foreseen.

This paper gives new insight into the advantages of adding bioceramics, such as HA and SrHA, to various composite materials for the purpose of bone regeneration. The results suggest that the biomimetic GelMA-SrHA composite hydrogel can provide a useful option for bone tissue engineering. 3D matrices provided an appropriate microenvironment for the development of cells. Both GelMA-HA and GelMA-SrHA are promising hydrogel composite solutions for bone tissue repair. The scaffolds were found to be suitable for the development of osteoblast cells in vitro. The composite material also proved to be printable into 3D constructs with good shape fidelity with respect to the 3D model. We demonstrated the suitability of a GelMA-HA bioink for 3D bioprinting of bone tissue engineering applications. The GelMA-based composite materials used in this study open new possibilities for other materials that can be utilized for bioprinting and bone defect site regeneration.

## Figures and Tables

**Table 1:** Sample composition computed from TGA.

Sample	GelMA (% wt.)	HA/SrHA (% wt.)
GelMA-HA HT	62.0	38.0
GelMA-HA HT-SR-10	63.1	36.9
GelMA-HA PR	62.6	37.4
GelMA-HA PR SR-10	67.4	32.6

## Data Availability

Data are contained within the article.

## Supplementary Materials

The following supporting information can be downloaded at https://www.mdpi.com/article/10.3390/polym16131932/s1. Figure S1. TEM images of HA/SrHA powders. Table S1. Feret diameter of HA/SrHA powders from TEM expressed in nm. Figure S2. Feret diameter histograms of HA/SrHA powders’ pore size distribution. TEM images were analyzed using ImageJ and data were computed using Excel software. Values are expressed in nm.
