# Supplementary material for: 3D-Bioprinted Gelatin Methacryloyl-Strontium-Doped Hydroxyapatite Composite Hydrogels Scaffolds for Bone Tissue Regeneration
Source: Polymers (Basel). 2024 Jul 6;16(13):1932. doi: 10.3390/polym16131932 (PMC11244251; doi:10.3390/polym16131932)
Supplement: Supplementary file 1 [file polymers-16-01932-s001.zip › polymers-3065957-supplementary.pdf]

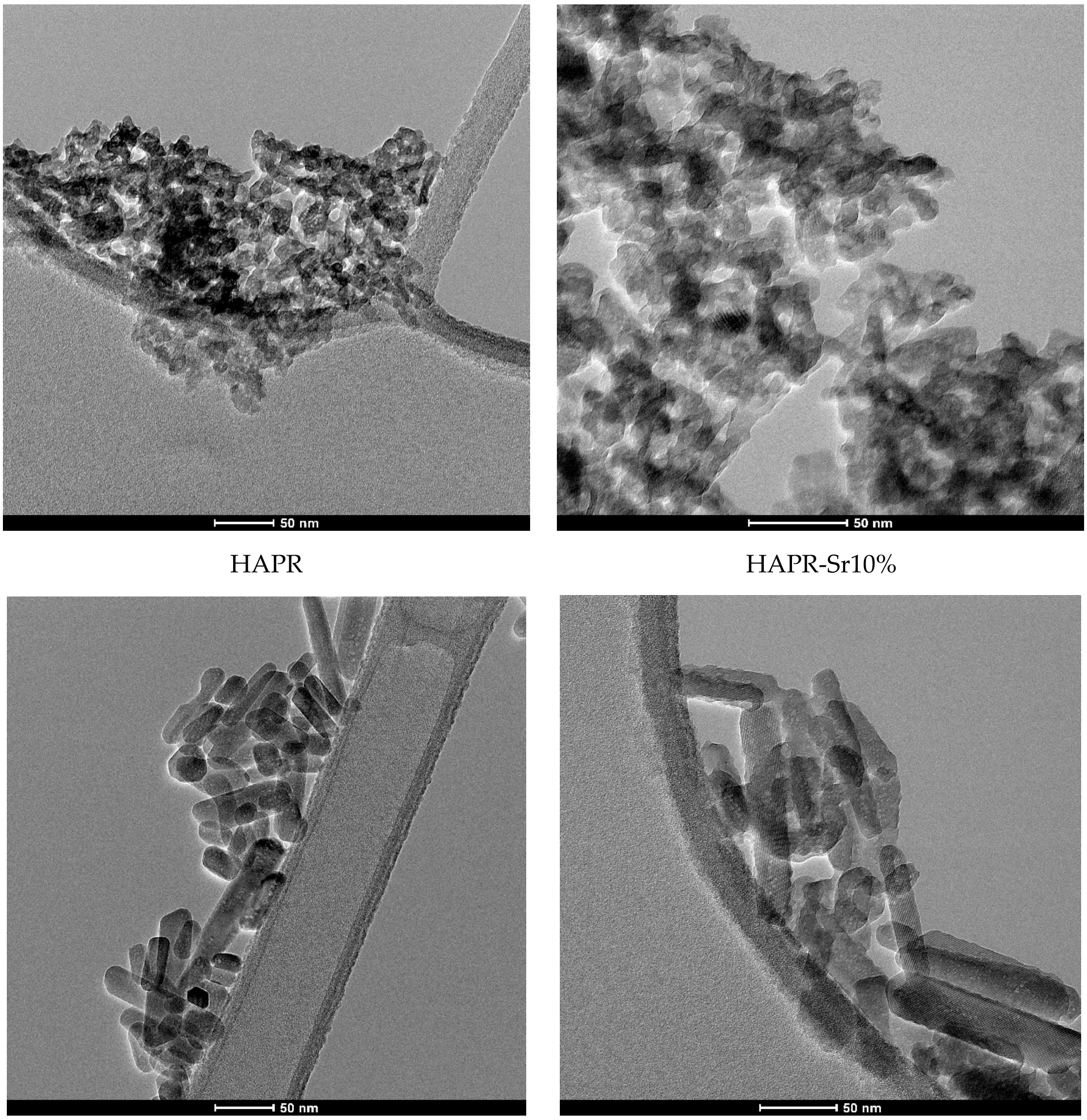

Figure S1. TEM images of HA/SrHA powders.

Table S1. Feret diameter of HA/SrHA powders from TEM expressed in nm.

Sample	Mean	SD	Min	Max
HA HT	57.323	32.468	25.356	123.264
HA HT-SR10%	75.59	29.751	35.132	148.377

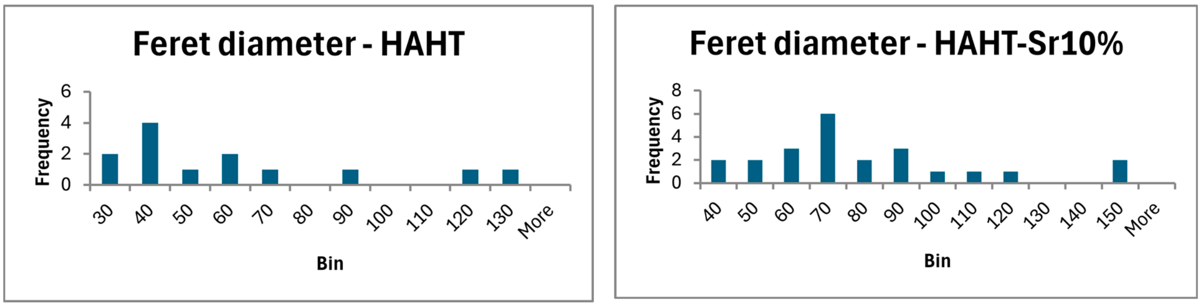

HAHT **HAHT-Sr10%**

Figure S2. Feret diameter histograms of HA/SrHA powders' pore size distribution. TEM images were analyzed using ImageJ and data were computed using Excel software. Values are expressed in nm.
